# The Eradication of *Helicobacter pylori* Was Significantly Associated with Compositional Patterns of Orointestinal Axis Microbiota

**DOI:** 10.3390/pathogens12060832

**Published:** 2023-06-15

**Authors:** Sally Ali Tawfik, Marwa Azab, Mohammed Ramadan, Sarah Shabayek, Ali Abdellah, Sultan S. Al Thagfan, Mohammed Salah

**Affiliations:** 1 Department of Microbiology and Immunology, Faculty of Pharmacy, Suez Canal University, Ismailia 41522, Egypt; 2 Department of Microbiology and Immunology, Faculty of Pharmacy, Al-Azhar University, Assiut 71524, Egypt; 3 Department of Clinical and Hospital Pharmacy, College of Pharmacy, Taibah University, Al Madinah Al Munaearah 42353, Saudi Arabia; 4 Department of Microbiology and Immunology, Faculty of Pharmacy, Port Said University, Port Said 42511, Egypt

**Keywords:** orointestinal axis, gut microbiota, *Helicobacter pylori* eradication, 16S rRNA gene

## Abstract

Background: *Helicobacter pylori* (*H. pylori*) is significantly linked to various diseases that seriously impact human health, such as gastric ulcers, chronic gastritis and gastric adenocarcinoma. Methods: The compositional shifts in bacterial communities of the orointestinal axis were surveyed pre/post-eradication of *H. pylori*. In total, 60 samples, including stool and salivary specimens, were collected from 15 *H. pylori*-positive individuals (HPP) before beginning and 2 months after receiving the eradication therapy. The V3-V4 regions of the 16S rRNA gene were sequenced using MiSeq. Results: Overall, oral microbiomes were collectively more diverse than the gut microbiomes (Kruskal–Wallis; *p* = 3.69 × 10^−5^). Notably, the eradication of *H. pylori* was associated with a significant reduction in the bacterial diversity along the orointestinal axis (Wilcoxon rank sum test; *p* = 6.38 × 10^−3^). Interestingly, the oral microbiome of HPP showed a positive correlation between *Proteobacteria* and *Fusobacteria,* in addition to a significant predominance of *Streptococcus*, in addition to *Eubacterium_eligens, Haemophilus, Ruminococcaceae, Actinomyces* and *Staphylococcus*. On the other hand, *Fusobacterium, Veillonella, Catenibacterium, Neisseria and Prevotella* were significantly enriched upon eradication *of H. pylori*. Generally, *Bacteroidetes* and *Fusobacteria* positively coexisted during *H. pylori* infection along the orointestinal axis (*r* = 0.67; *p* = 0.0006). The eradication of *H. pylori* was positively linked to two distinctive orotypes (O3 and O4). Orotype O4 was characterized by a robust abundance of *Veillonella* and *Fusobacteria*. The gut microbiomes during *H. pylori* infection showed a remarkable predominance of *Clostridium_sensu_stricto_1 and Escherichia_Shigella*. Likewise, *Bifidobacterium* and *Faecalibacterium* were significantly enriched upon eradication of *H. pylori*. Conclusions: Finally, the impact of eradication therapy clearly existed on the representation of certain genera, especially in the oral microbiome, which requires particular concern in order to counteract and limit their subsequent threats.

## 1. Introduction

*Helicobacter pylori* (*H. pylori*) is a gram-negative bacterium that is found in the gastric mucosa [1]. *H. pylori* is acquired during the early life stage, and the infection continues throughout the patients’ whole lives [2,3,4]. Half of the world’s population is infected with *H. pylori* which causes gastric diseases such as chronic atrophic gastritis, superficial gastritis and peptic and duodenal ulcers [5]. Moreover, diseases outside the gastrointestinal tract, such as iron deficiency anemia and idiopathic thrombocytopenic purpura, were found to occur due *to H. pylori* infection, which indicated a correlation between *H. pylori* infection and diseases outside the gastrointestinal tract [6,7]. International Agency for Research on Cancer has classified *H. pylori* as a class I carcinogen [8]. Several studies have revealed that the earlier the eradication of *H. pylori*, the lower will be the probability of gastric cancer [9,10]. That’s why, in some countries, such as Japan and Korea, nationwide eradication therapy has been applied. This is considered as a primary prevention method to treat gastric cancer disease [11,12].

The majority of oral bacteria species are not easily cultured in vitro via traditional cultivation methods, but it requires advanced high-throughput molecular techniques [13]. It has been reported that *H. pylori* was found in the oral cavity as a result of stomach reflux. The oral cavity acts as a reservoir for *H. pylori* bacteria [14], and there are various routes for *H. pylori* transmission between individuals which include both gastro-oral and fecal-oral methods in contaminated food and water [14]. Regarding the oral and gut microbiota, it might originate from different parts of the digestive tract, which includes the bacterial passage from the oral cavity and esophagus to colonize the gastric mucosa or through reflux from the duodenum. There was a microbial similarity detected between gastric juice and the oral cavity [15].

*H. pylori* infection has been detected to result in dysbiosis of gastric and intestinal microbiota, which is a factor in diseases associated with *H. pylori* [16]. However, to state whether *H. pylori* infection is more harmful than useful for gastrointestinal microbiota is yet unclear [17]. Various studies have evaluated the influence of *H. pylori* eradication on the gut microbiome. After the *H. pylori* eradication treatment, it was discovered that the bacterial diversity decreased significantly. Therefore, it is important to investigate the impacts of eradication treatment changes on the gut microbiota composition to ensure the maintenance of microbiome homeostasis [18,19,20,21]. Interestingly, one study found that gut microbiota perturbations in several subjects remained for up to 4 years after eradication treatment [22]. The gut microbiota plays an essential role concerning human health, and any imbalance of this microbiota is linked to different diseases [13,14].

The *H. pylori* eradication effect on the oral and gut microbiota of adults is still not completely investigated. There were few studies investigating *H. pylori’s* relationship with the microbial community in the oral microbiome, although it was discovered that it passes via saliva [14]. Furthermore, the compositional shifts in the orointestinal axis microbiome that accompany the eradication of H. pylori are not significantly explored yet. Thus, this study targeted to dissect the underlying changes in the orointestinal axis microbiome upon eradication treatment.

## 2. Material and Methods

### 2.1. Ethics Statement

This study was approved by the Ethics Committee at the Faculty of Pharmacy, Suez Canal University, Egypt (reference number: 202009PHDH1). The present study was performed following the principles of the Declaration of Helsinki. Written informed consent was obtained from the patients involved in the study.

### 2.2. Patient Recruitment and Sample Collection

Participants were enrolled at outpatient clinics of the Internal Medicine Department, Al Mabarrah Health Insurance Hospital, Zagazig, Egypt. Patients were diagnosed as *H. pylori*-positive individuals (HPP) by positive *H. pylori* stool antigen test and urea breath test (C13-UBT) [23]. The successful eradication of *H. pylori* was confirmed by a negative *H. pylori* stool antigen test 2 months after completing the eradication treatment [18]. Two stool samples and 2 saliva specimens were collected from participants upon HPP diagnosis and 2 months after receiving the eradication therapy [24]. The experimental design was based on comparisons of our 4 study groups (Supplementary Figure S1).

The included subjects had good oral hygiene in such a way that they regularly brushed their teeth twice daily for 3 minutes each time [14]. Patients with any of the following criteria were excluded from this study: (1) previous eradication treatment, (2) allergy to any drugs included in this study, (3) previous gastric surgery, (4) the coexistence of severe concomitant illness, (5) pregnant or lactating women, (6) antibiotics, proton pump inhibitor, or probiotics administration during the past 8 weeks, (7) the existence of dental carious and oral abscesses, (8) the presence of any untreated cavitated carious lesions, (9) periodontal disease or periodontal pockets ≥ 4 mm, (10) patients suffering from any systemic diseases such as hypertension, diabetes, or cardiopathy, (11) smoker patients [14,18,25]. The recruited patients received a triple eradication regimen which included clarithromycin (500 mg), omeprazole (20 mg) and amoxicillin (500 mg) for 14 days [25].

Stool samples were collected freshly in a sterile stool plastic container, while saliva samples were collected using a sterile cotton oral swab. Saliva sample collection was carried out in the morning 2 hours after eating [14].

### 2.3. Genomic DNA Extraction

Genomic DNA of saliva and stool samples were extracted using a Qiagen DNeasy power soil kit (cat. No 12888-100) as recommended by the manufacturer. Oral and stool samples were evaluated by measuring the samples’ absorbance values at 260 and 280 nm. This was performed using a Nanodrop ND-1000 spectrophotometer (ND-1000; Thermo Scientific, Waltham, MA, USA) [26].

### 2.4. Polymerase Chain Reaction (PCR) Amplification and Sequencing of the 16S rRNA Amplicon

The extracted DNA was amplified by PCR targeting the hypervariable region (V3-V4) of the bacterial 16S rRNA. The V3-V4 hypervariable region of the 16S rRNA gene was amplified using Forward Primer 5′ TCGTCGGCAGCGTCAGATGTGTATAAGAGACAGCCTACGGGNGGCWGCAG and Reverse Primer 5 GTCTCGTGGGCTCGGAGATGTGTATAAGAGACAGGACTACHVGGGTATCTAATCC. The Illumina adapters in those primers are underlined [26]. Sequencing of PCR amplicons was performed at IGA Technology Services (Udine, Italy) on an Illumina MiSeq platform according to the manufacturer’s instructions (Illumina, San Diego, CA, USA) [26,27].

### 2.5. Bioinformatics and Data Analysis of 16S rRNA Gene Sequencing

The preprocessing of raw 16S rRNA sequences was performed by inputting raw reads in the Quantitative Insights Into Microbial Ecology 2 platform (QIIME2) [28]. The classification of 16S rRNA reads to representative reads was based on Amplicon Sequence Variants (ASVs). Trimming and filtering out, denoising and outputting high-resolution representative ASVs were achieved using DADA2 plugged in QIIME2 (Phred quality ≥19, maximum of two expected errors per read = 2, truncation length 270 and 210 for forward and reverse reads, respectively) [29]. Pre-trained RDP’s naive Bayesian classifier was employed for the taxonomy assignment of ASVs against SILVA reference sequences (V138) [30] at 97% sequence similarity [31].

QIIME2 scripts were run on the entire dataset to identify the patterns of microbial diversity that were associated with the orointestinal axis microbiome. The statistical significance of shifts in bacterial diversity along the orointestinal axis was elucidated using the nonparametric Wilcoxon rank-sum test and the Kruskal–Wallis rank-sum test. The resulting *p*-values were adjusted using the false discovery rate method (FDR) [32]. Inter-community features (alpha diversity) were identified using observed species and the Shannon diversity index. The similarities between the studied microbiomes were assessed by applying unweighted and weighted UniFrac distance matrices on the generated ASVs. The significance of the clustering of the studied groups was assessed using Permutational Multivariate Analysis of Variance (PRERMANOVA)(Adonis R, package Vegan) [33].

To define the differentially represented genera that drove the shifts in microbiomes, DESeq2 was applied to all genera in the dataset (FDR-corrected *p*-value < 0.05) [34]. The Correlations between bacterial taxa at different taxonomic levels were assessed by applying Spearman correlation distance (r ≥ ±0.6, *p* ≤ 0.05) on the dominant taxa (mean relative abundance ≥1.37; R package, Hamsic) [35]. Furthermore, the enterotyping approach was employed to identify the genera that stratify the microbiomes into distinct clusters [36]. Likewise, the taxon that was detected in all of the samples belonging to one site was considered a core taxon for that site. The linear discriminant analysis (LDA) effective size (LEfSe) was performed to define the potential biomarkers associated with each site at sampling (LDA scores >3.0, α = 0.05) [37].

### 2.6. Data Availability

This study of 16S rRNA raw sequences has been submitted in NCBI bioproject under accession number PRJNA901020 (http://www.ncbi.nlm.nih.gov/bioproject/901020 accessed on 11 June 2023) and biosamples (SAMN31721358:SAMN31721717) and accessed on 11 June 2023. The sequences are available at the NCBI Sequence Read Archive (SRA) under accession numbers (SRR22293484:SRR22293543).

## 3. Results

### 3.1. Characteristics of Patients and Data

In total, sixty saliva and stool specimens were derived from 15 *H. pylori*-positive adults pre/post eradication of *H. pylori* (eight females and seven males; age: mean ± SD; 47.2 ± 15.15 years). Overall, 2,424,317 raw reads were inputted to Qiime2 and generated 1,912,721 reads (78.89% of all raw reads; median length = 446 bp) which were obtained by merging forward and reverses reads, quality checking, removing low-quality reads and potential chimeric sequences that were used in downstream analyses.

### 3.2. Distinct Taxonomic Profiles Accompany the Pre/Post-Eradication of H. pylori Infection along the Orointestinal Axis

**Figure 1:**
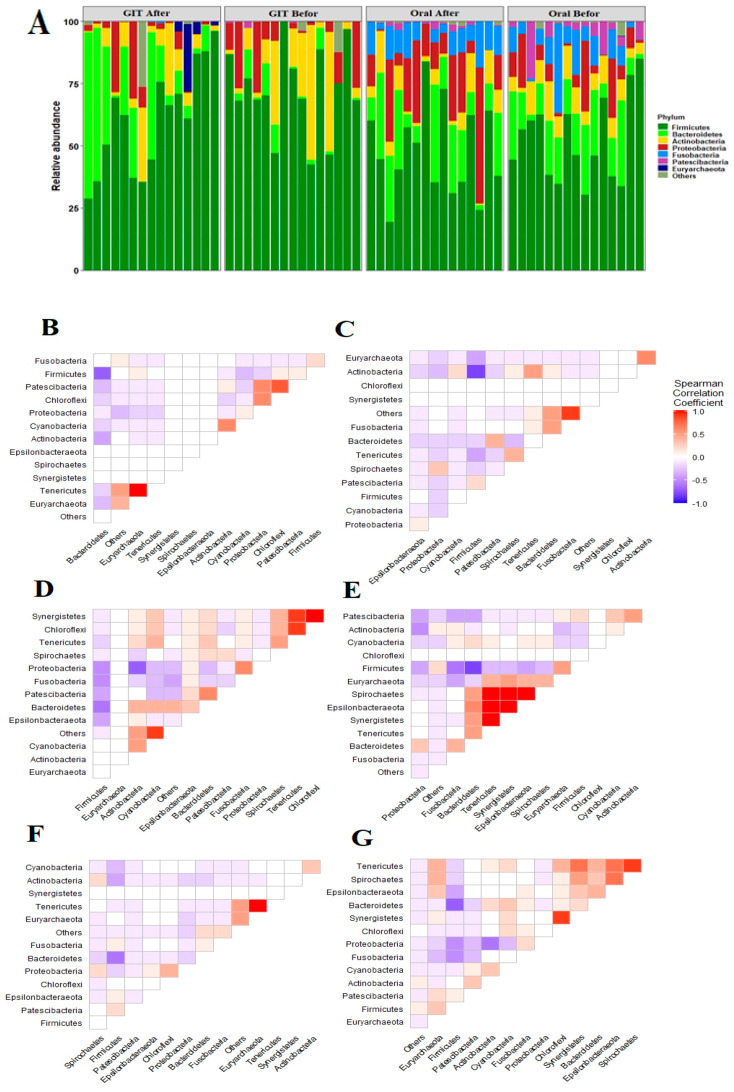
Phylum level analysis of oral and gut microbiota. (**A**) Bar charts illustrated the relative abundance of the major phyla in the oral and gut microbiome before and after eradication therapy of subjects. The *X*-axis represents the relative abundance. The Y- axis defined the study groups. Corrplots, based on the Spearman correlation coefficient, highlight the association between the main phyla: (**B**,**C**) represent the association between dominant phyla in oral microbiomes pre/post eradication of *H. pylori*. (**D**,**E**) define the correlation between the main phyla of gut microbiomes before and after receiving the treatment. (**F**,**G**) illustrate the association between the major phyla in the microbiomes of the orointestinal axis during and after the eradication of *H. pylori*.

In total, 8846 ASVs were assigned to the corresponding taxonomy in the SILVA database. The overall taxonomy profile of the studied groups was composed of 26 phyla, 42 classes, 97 orders, 247 families, 643 genera and 1358 species. The most abundant phyla across the oral specimens were *Firmicutes* (50.4%), *Bacteroidetes* (16.9%), *Proteobacteria* (12.6%), *Fusobacteria* (8.8%), *Actinobacteria* (7.4%) and *Patescibacteria* (3.3%) (Figure 1A). Regarding stool samples, *Firmicutes* showed significantly variable proportions in relation to the treatment (68.28% and 47.98% for pre- and post-eradication therapy, respectively; Kruskal–Wallis: *p =* 6.17 × 10^−4^). In addition, *Proteobacteria* and *Actinobacteria* were enriched during *H. pylori* infections (13.91% and 9.57%, respectively) in comparison to the post-eradication period (7.52 and 5.82%, respectively).

Interestingly, *Proteobacteria* and *Bacteroidetes* showed variable proportions between either the two sites or sampling events, whereas *Proteobacteria* in the oral microbiome existed with a relatively diminished abundance before the treatment and was subsequently overrepresented upon eradication of *H. pylori. Bacteroidetes* was in contrast to *Proteobacteria* in both sites with enriched proportion in gut microbiome upon eradication of *H. pylori*. Furthermore, *Bacteroidetes* and *Fusobacteria* were positively correlated during *H. pylori* infection along the orointestinal axis (*r* = 0.67; *p* = 0.0006). Remarkably, *Firmicutes* and *Bacteroidetes* negatively coexisted in all the studied groups and along the orointestinal axis (Figure 1).

### 3.3. H. pylori Infections Are Positively Linked with Diverse Orointestinal axis Microbiomes

**Figure 2:**
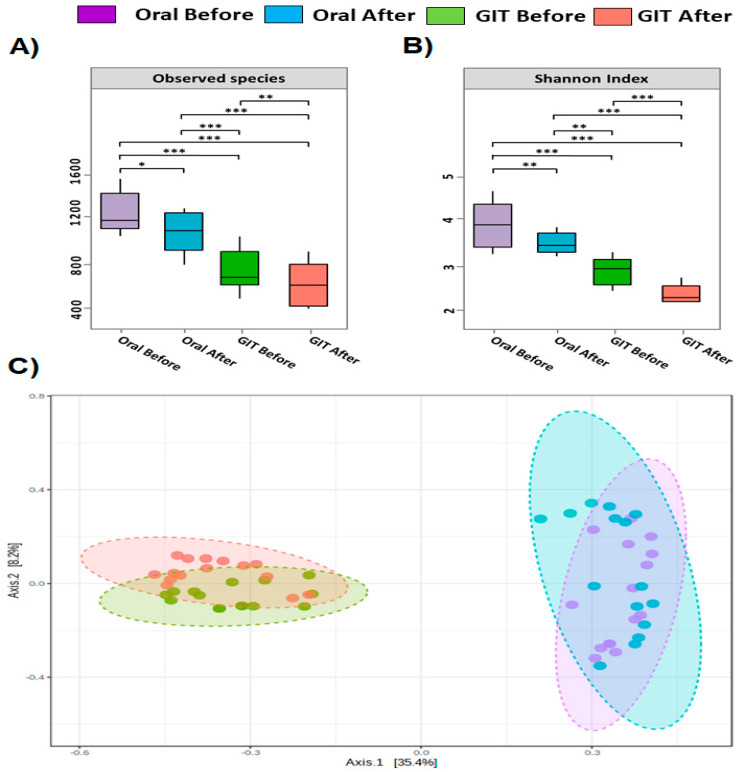
Bacterial diversity measurements of orointestinal axis microbiomes. Alpha diversity indices of gut microbiomes illustrated in (**A**) and (**B**) panels: Box plots define the bacterial diversity in terms of richness (the number of observed species) and evenness (Shannon diversity index). The *X*-axis defines the study groups, and the *Y*-axis denotes the alpha diversity indices. The line in each box indicates the median, the boxes define the interquartile range (IQR) between the 25th and 75th percentile, and the whisker delimits the range. The nonparametric Wilcoxon rank-sum test was employed to identify the statistical significance of pairwise comparisons. Significant differences were indicated with either * (*p* < 0.05), ** (*p* < 0.01) or *** (*p* < 0.001). (**C**) panel represented PCoA two-dimensional graph of oral and gut microbial community membership before and after eradication therapy (axis 1 = 35.4%; axis 2 = 8.2%). Colors represented the four groups based on before and after treatment. Purple- and blue-colored balls resembled oral samples before and after treatment, respectively. Green- and pink-colored balls resembled stool samples before and after treatment, respectively. Ellipses indicate significant clustering according to site and treatment at (*p*-value < 0.001, PERMANOVA).

Bacterial diversity analyses in terms of alpha and beta diversity were measured using the entire dataset (Figure 2). Of note, the eradication of *H. pylori* exhibited a distinct reduction in the bacterial diversity along the orointestinal axis in contrast to HPP (Kruskal–Wallis; *p* = 3.69 × 10^−5^; Figure 2A,B). Also, oral microbiota was significantly more diverse than gut microbiomes regardless of the *H. pylori* infection (Wilcoxon rank sum test; *p* = 6.38 × 10^−3^). Furthermore, the overall taxonomic composition showed anatomical and disease-based clustering of samples (Figure 2C; PERMANOVA; 13.728; R-squared: 0.42378; *p*-value: 0.001).

### 3.4. Orointestinal Axis Microbiomes Have Potential Biomarkers and Microbial Signatures for Pre/Post Eradication of H. pylori

**Figure 3:**
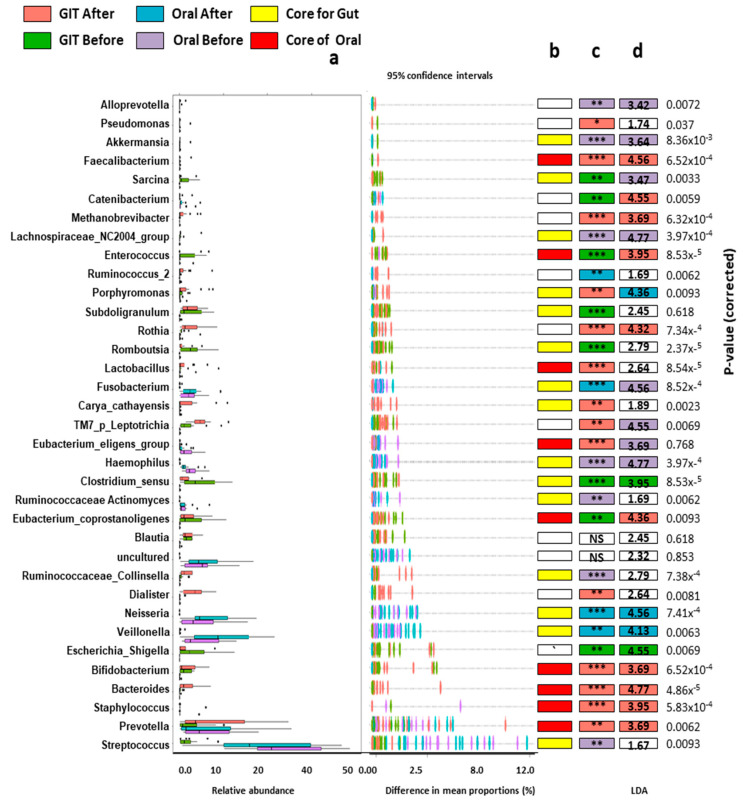
The genus level-based analysis of gut microbiomes associated with the orointestinal axis. (**a**) Box plots denote the relative abundance and differences in mean proportions with 95% confidence intervals. (**b**) The colored boxes describe the core genera of gut and oral microbiomes. (**c**) DESEQ2 was implemented to identify the significantly represented genera between the studied groups. The significant differences were represented either * (*p* < 0.05), ** (*p* < 0.01) or *** (*p* < 0.001). (**d**) LEfSe was performed to define the Candidate biomarkers for the studied groups, and the numbers indicate LDA scores.

Differentially abundant genera were identified by applying DESEQ2 to all genera in the studied samples (Figure 3). The orointestinal axis microbiomes before the treatment were characterized by the over-representation and positive coexistence of *Staphylococcus* and *Enterococcus* (*r =* 0.086; *p* = 0.001). On the other hand, the eradication of *H. pylori* was accompanied by an overrepresentation of *Rothia, Romboutsia, Lactobacillus, TM7_p_Lleptotichia, Clostridium_sensu, Blautia, Eubacterium_coprostangolins* and *Bifidobacterium*. Interestingly, the saliva and gut microbiomes were manifested with apparent proportions of certain genera, which could be considered as a biological signature (biomarker) for either the site or the treatment. Regarding oral microbiomes, the *H. pylori* infection was distinguished by an over predominance of *Eubacterium_eligens, Ruminococcaceae_Actinomyces, Enterococcus, Haemophilus* and *Staphylococcus*. The post-eradication of *H. pylori* showed an enriched abundance of *Catenibacterium*, *Rothia*, *Lactobacillus*, *Fusobacterium* and *Veillonella*, in addition to the notable predominance of *Prevotella*.

On the other side, the gut microbiome of HPP displayed a prominent representation of *Clostridium_sensu_stricto_1, Pseudomonas, Sarcina, Romboutsia, Lactobacillus, Eubacterium_ coprostangolins, Blautia, Bifidobacterium and Streptococcus. On the contrary, Faecalibacterium, Catenibacterium, Methanobrevibacter, Porphyromonas, Rothia, Carya_cathayensis, Dialiaster, Bacteroides* and *Prevotella* increased in abundance after eradication.

### 3.5. Enterotype and Orotypes of Pre/Post H. pylori Infection

To define the main driver of the orointestinal axis, enterotype analysis was applied to all samples. The term orotype was introduced as being synonymous with the oral cluster. The dominance of *Streptococcus, Prevotella*, *Neisseria, Clostridium_sensu_stricto_1* and *Viellonella* distinctly clustered the microbiomes to sex enterotypes (Table 1). Oral microbiomes showed a remarkable clustering of samples to four orotypes that mainly were based on the classification of pre/post-eradication of *H. pylori* to two orotypes for each event.

### 3.6. Bidirectional Association between Microbiomes, Either Anatomical Sites or the Disease State

**Figure 4:**
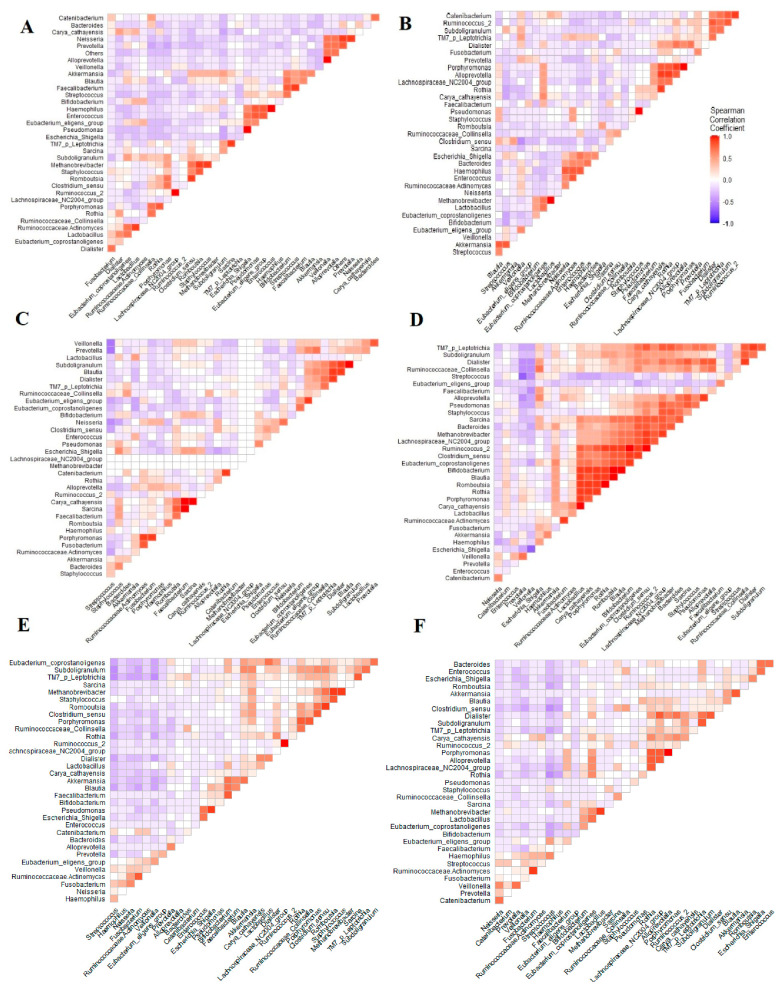
Corrplots based on the Spearman correlation coefficient highlighting the association between the dominant genera: (**A**,**B**) demonstrate the association between the dominant genera in oral microbiomes pre/post eradication of *H. pylori*. (**C**,**D**) define the correlation between the main genera of gut microbiomes before and after receiving the treatment. (**E**,**F**) represent the association between the major genera in the microbiomes of the orointestinal axis during and after the eradication of *H. pylori*.

Correlations between oral and gut microbiome before and after eradication at genus taxonomic levels were assessed to infer the coexistence of the dominant genera at the two sites and along the orointestinal axis (Figure 4). Regarding the orointestinal axis, fusobacterium was negatively correlated to *Clostridium_sensu_stricto_1.* Oppositely, *Streptococcus* was positively correlated to *Clostridium_sensu_stricto_1.* Interestingly, combining the oral and saliva microbiomes in pre/post-*H. pylori* infection revealed that *Clostridium_sensu_stricto_1* was significantly linked to a cluster of genera, including *Romboutsia*, *Porphyromonas* and *Rothia*.

## 4. Discussion

*Helicobacter pylori* (*H. pylori*) is significantly linked to various diseases that seriously impact human health, such as gastric ulcer, chronic gastritis and gastric adenocarcinoma. *H. pylori* is frequently accompanied by pathophysiological perturbations in the gastric environment, which in turn reshape the microbiota of the orointestinal axis. In this study, we carried out a self-comparison study before and after eradication treatment of salivary and fecal samples to compare oral and gut microbiome composition at the same point in time. It has been shown in a few studies that *H. pylori* can be transmitted by oral-oral or fecal-oral methods [38]. It was detected that sharing of food, tableware and even kisses could play a role in the transmission of *H. pylori*. There were a number of previous studies that revealed the existence of *H. pylori* in saliva [39]. Since those bacteria found in the plaque adhere to the gums, therefore, it is more logical to examine the saliva rather than examining the dental plaque in this study. Previous research has suggested the benefits of *H. pylori* eradication to prevent gastric cancer and the recrudescence of peptic ulcers. Thus, the impact of antibiotics and proton pump inhibitors on the gut microbiome has focused more attention [9,25].

To date, the *H. pylori* eradication therapy regimen involves dual therapy, triple therapy and quadruple therapy [40,41]. Triple therapy stays the standard guideline of the European *Helicobacter* and Microbiota Study Group in areas where clarithromycin resistance is low [42]. EGené et al. study revealed that triple and quadruple therapies were nearly equivalent in effectiveness, compliance and even side effects when administrated to patients suffering from *H. pylori* infection as a first line of treatment [43]. The administration of triple therapy and its rate of success ranges from 80–85% [44]. Furthermore, the eradication therapy of *H. pylori* was recently linked to short-term and long-term (less than 6 months) alterations in the gut microbiota of treated individuals (52.3% of cases) [45,46].

The oral samples demonstrated that the relative abundance of *Firmicutes, Actinobacteria* and *Patescibacteria* declined following treatment while the relative abundance of *Bacteroidetes*, *Fusobacteria* and *Proteobacteria* increased. On the other hand, regarding stool samples which elucidated that the relative abundance of *Firmicutes, Actinobacteria, Proteobacteria, Verrucomicrobia* and *Patescibacteria* decreased after treatment, whereas the relative abundance of *Bacteroidetes*, *Euryarchaeota* and *Cyanobacteria* increased. Gao et al. [16] and Hold and Hansen [47] reported in their studies that *H. pylori* infection lowered colon acidity leading to a decrease in the relative abundance of *Bacteroidetes* while *Firmicutes* and *Proteobacteria* increased in their relative abundance. Their results were in line with our current study. Various studies demonstrated that *H. pylori* infection lowers the *Actinobacteria* abundance [48], as represented in our study, but on the contrary, He et al. [17] reported an increase.

Although *Proteobacteria* is regarded as a natural inhabitant of the intestine, it constitutes a small part of a healthy microbiome [25]. There was a conflict in the results of the relative abundance of *Proteobacteria* in salivary and fecal samples in the collected samples, as salivary samples showed an enriched abundance of *Proteobacteria* after eradication therapy while fecal samples revealed a decrease in their relative abundance. The increase in the *Proteobacteria* relative abundance in oral samples after eradication therapy might be due to the inhibitory effect of *H. pylori* eradication on commensal bacteria. Some studies suggested that increased *Proteobacteria* prevalence in microbial communities can be a possible sign of risk disease and dysbiosis [49].

Overall, oral microbiomes were collectively more diverse than the gut microbiomes. Notably, the eradication of *H. pylori* was associated with a significant reduction in bacterial diversity along the orointestinal axis. This finding was consistent with previous studies that reported a notable reduction in bacterial diversity upon eradication of *H. pylori* which could be attributed to the impact of reshaping the gut microbiome as a result of the eradication of *H. pylori* and the used therapy such as proton pump inhibitor and antibiotics which in turn could manipulate the oral microbiome [14,17,18,19,22,25,27,48,50,51,52,53,54].

Furthermore, the anatomical site-dependent compositional and structural patterns of orointestinal axis microbiomes were clearly manifested in the current study. Beta diversity analysis based on the Bray–Curtis index indicated that the community structure of salivary microbiota before and after eradication therapy was different, and this observation was consistent with Ji et al. finding [14]. Both salivary and fecal samples pre and post-treatment appeared to cluster according to treatment and site, suggesting that *H. pylori* infection may affect oral and GIT bacterial communities. Obvious separation by the PCoA and PERMANOVA analysis before and after *H. pylori* eradication therapy clarified that eradication therapy altered the oral and gut microbiome to some extent.

*Porphyromonas gingivalis* (*P. gingivalis*) is one of the bacteria which plays a role in gut diseases and periodontitis in the oral cavity. *P. gingivalis* induced the tight junction proteins depression causing changes in the gut microbiota [55]. In our findings, *P. gingivalis* was found to be overrepresented after eradication therapy in oral and stool samples which means that *H. pylori* greatly affects its existence. *P. gingivalis* was closely related to dysbiosis of the oral-gut axis microbiome. *H. pylori* eradication was associated with *Porphyromonas* overrepresentation which must be taken into consideration.

*Staphylococcus aureus* (*S. aureus*) colonizes the anterior nares [56], that’s why their existence in the oral cavity is reasonable but at the same time controversial [57]. It is not obvious whether *S. aureus* plays a portion in the ecology of the healthy normal oral flora or not [58]. Various research has indicated that the oral cavity acts as a reservoir for *S. aureus* in immunosuppressed patients [57]. *S. aureus* infections may cause oral diseases like periodontitis and dental caries [59]. Not only this, but it might also cause systemic diseases such as chronic kidney disease, heart disease, Crohn’s disease and orofacial granulomatosis [60,61,62]. In addition, oral *S. aureus* has been observed as a causative factor in infective endocarditis [63]. Interestingly, *S. aureus* relative abundance in our study decreased after eradication treatment indicating the influence of eradication on the oral microbiome and the shift in the community composition.

*Streptococcus, Enterococcus* and *Bifidobacterium* are probiotics found in the gastrointestinal tract which provide health benefits to the host [64,65,66,67]. Safe strains of *Enterococcus *are regarded as probiotics in the human gut [68]. These genera were found to have lower abundance in the orointestinal axis after eradication therapy. Overall, *H. pylori* eradication was revealed to affect and change the abundance of the previously mentioned probiotics in the Oro-intestinal axis microbiome. Previous studies have explained that probiotics supplementation can reduce the changes and imbalance in the gut microbiome due to antibiotics [69,70]. Probiotics supplementation in eradication therapy can decrease dysbiosis and the adverse effects of treatment [18].

The metabolites of the bacteria perform the function of signaling between the gut microbiome and the host. From these bacterial metabolites, there are short-chain fatty acids (SCFAs) which are regarded as the most abundant microbial metabolites. SCFAs are generated by anaerobic gut bacteria through the fermentation of complex carbohydrates which weren’t digested and absorbed in the small intestine [71]. The bacteria that belong to the *Firmicutes* phylum are able to ferment carbohydrates into SCFAs that increase the intestinal barrier function [72]. There are several functions of these metabolites, such as increasing the junctions’ level in the gastrointestinal tract, preventing inflammations and decreasing mucosal permeability, which are triggered through cytokines [73]. The decrease in the level of SCFAs is related to changes in the host’s health and metabolism [74].

We observed an increase in *Bacteroides* and *Faecalibacterium* genera post*-H. pylori* eradication in stool samples which are categorized as bacteria-producing SCFAs. *Bacteroides* genera are considered a dominant member of the gut microbiome which participates in the metabolism of carbohydrates and the synthesis of conjugated linoleic acid. In addition, it exerts anti-atherogenic, anti-diabetic, anti-obesogenic and anti-hyperlipidemic effects as well as immunomodulatory properties [75]. Another genus that is highly abundant in the healthy individual colon is the *Faecalibacterium* genus which plays an important role in producing energy for colonocytes and mediating anti-inflammatory action [23,76]. Lin et al. study [77] stated that *Faecalibacterium* produces butyrate, which is one of the main short-chain fatty acids found in the gut microbiota. It was found that patients with metabolic and intestinal disorders (for instance, irritable bowel syndrome and inflammatory bowel disease) had decreased levels of the *Faecalibacterium* genus, which could be considered a biomarker of a healthy intestine [76,78,79]. Thus, *Bacteroides* and *Faecalibacterium* genera are considered beneficial bacteria [25]. In this line of thought, we could conclude that the higher abundance of *Bacteroides* and *Faecalibacterium* post-eradication was due to compensatory and protective mechanisms.

On the other hand, there are detrimental bacteria as well in the GIT, such as *Escherichia*-*Shigella.* However, *Escherichia-Shigella* relative abundance was increased during *H. pylori* infection. The overgrowth of *Escherichia-Shigella* can be associated with dysbiosis of the gut flora leading to serious diseases such as non-alcoholic fatty liver disease, acute necrotizing pancreatitis and inflammatory bowel disease [80,81]. Added to this, *Escherichia-Shigella* are resistant to a broad range of antibiotics which can spread genes of resistance to both commensal and pathogenic bacteria through horizontal gene transfer [82,83].

*Prevotella* is a common colonizer of the oral-gut microbiome [84]. This study reported that *Prevotella* genus relative abundance increased in association with *H. pylori* eradication leading to orointestinal axis microbiota remodeling. The *H. pylori* infection affected the growth of *Prevotella,* but the mechanism still needed to be explored.

*Clostridium_sensu_stricto*_1 is an opportunistic pathogen that causes intestinal inflammation and lowers the content of SCFAs [85]. In our study, we found that *Clostridium_sensu_stricto*_1 negatively correlated with *fusobacterium* and SCFAs, and positively correlated with *streptococcus* during *H. pylori* infection, which indicated that it might be concerned with the manifestation of changes and shifts in the orointestinal axis microbiota. *Clostridium_sensu_stricto*_1 was found to be overrepresented during *H. pylori* that might occur as a result of compensatory mechanisms.

Our result concerning oral samples showed an augment in *Fusobacterium* abundance after *H. pylori* eradication. This genus is pathogenic bacteria that cause infective endocarditis, pneumonia, systemic infections, liver abscess and meningitis [86,87]. It was stated that an increase in *Fusobacterium* abundance was associated with oral cancer and colorectal cancer [88,89]. Lavelle and Sokol’s study as well indicated that *Fusobacterium* increased in patients suffering from irritable bowel syndrome (IBS) and was rarely found in healthy persons [90]. Added to this, it can trigger colitis through disruption of the epithelial barrier and results in causing inflammation [91]. It is worth mentioning that elevated *Fusobacterium* abundance post-eradication may be related to higher oral cancer risk. Therefore, monitoring *Fusobacterium* relative abundance post*-H. pylori* eradication is to be considered necessary.

Interestingly, the *Veillonella* genus was detected to be overrepresented post*-H. pylori* eradication in salivary samples. *Veillonella* causes oral diseases such as periodontic and endodontic diseases in humans [92]. Added to this, *Veillonella* plays a critical role in atherosclerosis and cardiovascular disease development [93]. Thus, it is essential to take into account the elevated abundance level of *Veillonella* after eradication and its possible effects on human health.

This is the first study in Egypt and the Middle East to demonstrate oral and gut microbiota alterations defining the orointestinal axis as a result of *H. pylori* infection in a group of patients to investigate the relationship between two different sites before and after eradication therapy. Our results indicated that eradication of *H. pylori* infection could change the salivary and gut microbiota composition. To the best of our knowledge, this is the premier prospective study to illustrate *H. pylori*‘s post-eradication impact on orointestinal axis microbiota in Egyptian individuals. On the other hand, the limitations of the present study include the slightly limited sample size and tracking the patterns of orointestinal axis microbiomes at different periods after the eradication of *H. pylori* to infer the required time for recovery of microbiomes. Also, exploring the overall functional potential of the orointestinal axis microbiome during and after the eradication of *H. pylori* might provide evidence about the crosstalk between the microbiome and *H. pylori*.

## 5. Conclusions

*H. pylori* is positively associated with collective shifts in microbiomes along the orointestinal axis, which were obviously reshaped upon receiving eradication therapy. The microbiomes along the orointestinal axis during *H. pylori* infections potentially either protect or implicate the underlying protective or pathogenic mechanisms. Furthermore, the overall intra-microbial relations potentially counteract the predominance of pathogenic residents, in addition to compensatory and recovery mechanisms against the subsequent harmful pathophysiological impacts of *H. pylori* and the dominant pathogenic competitors. The eradication of *H. pylori* results in substantial changes in the oral and gut microbiome, which resulted in the robust predominance of harmful bacteria such as *Fusobacterium*. Finally, the present study provides new insight into the shifts in the oral microbiome that accompany the eradication of *H. pylori* and an alert about the required interventions in order to frustrate the enrichment of pathogenic bacteria during post*-H. pylori* transience. Further future prospective investigations and sequencing of the whole metagenome might be required to provide a deeper insight into the taxonomic and functional profile of the oral and gut microbiota.

## Figures and Tables

**Table 1:** Enterotypes of Oro-intestinal axis microbiomes.

Oral Before		Oral After		Gut Before	Gut After
O1	O2	O3	O4	G1	G2
*Streptococcus*	*Streptococcus*	*Streptococcus*	*Neisseria*	*Clostridium_sensu_stricto_1*	*Prevotella*
*Neisseria*	*Fusobacterium*	*Neisseria*	*Prevotella*	*Escherichia_Shigella*	*Bacteroides*
*Ruminococcaceae Actinomyces*	*Haemophilus*	*Fusobacterium*	*Veillonella*	*Bifidobacterium*	*Bifidobacterium*
*Eubacterium_eligens_group*	*Enterococcus*	*Prevotella*	*Fusobacterium*	*Enterococcus*	*Lactobacillus*
*Porphyromonas*	*Neisseria*	*Rothia*	*Streptococcus*	*Subdoligranulum*	*Romboutsia*
*Prevotella*	*Prevotella*	*Ruminococcaceae Actinomyces*	*Ruminococcaceae Actinomyces*	*Ruminococcaceae_Collinsella*	*Sarcina*
*Bifidobacterium*	*Veillonella*	*Streptococcus*	*Staphylococcus*	*Prevotella*	*Escherichia_Shigella*
*Veillonella*	*Staphylococcus*		*Haemophilus*	*Eubacterium_coprostanoligenes*	*Blautia*
*Staphylococcus*				*Romboutsia*	

## Data Availability

This study of 16S rRNA raw sequences have been submitted in NCBI bioproject under accession number PRJNA901020 (http://www.ncbi.nlm.nih.gov/bioproject/901020) and biosamples (SAMN31721358:SAMN31721717) and accessed on 11 June 2023. The sequences are available at the NCBI Sequence Read Archive (SRA) under accession numbers (SRR22293484:SRR22293543).

## Supplementary Materials

The following supporting information can be downloaded at: https://www.mdpi.com/article/10.3390/pathogens12060832/s1: The study design. A total of 60 salivary and stool samples collected from 15 participants; 15 oral samples before eradication (OB); 15 oral samples after eradication (OA); 15 stool samples before eradication (SB); 15 stool samples after eradication (SA).
