# Supplementary material for: The Eradication of Helicobacter pylori Was Significantly Associated with Compositional Patterns of Orointestinal Axis Microbiota
Source: Pathogens. 2023 Jun 15;12(6):832. doi: 10.3390/pathogens12060832 (PMC10303999; doi:10.3390/pathogens12060832)
Supplement: Supplementary file 1 [file pathogens-12-00832-s001.zip › pathogens-2418609-supplementary.pdf]

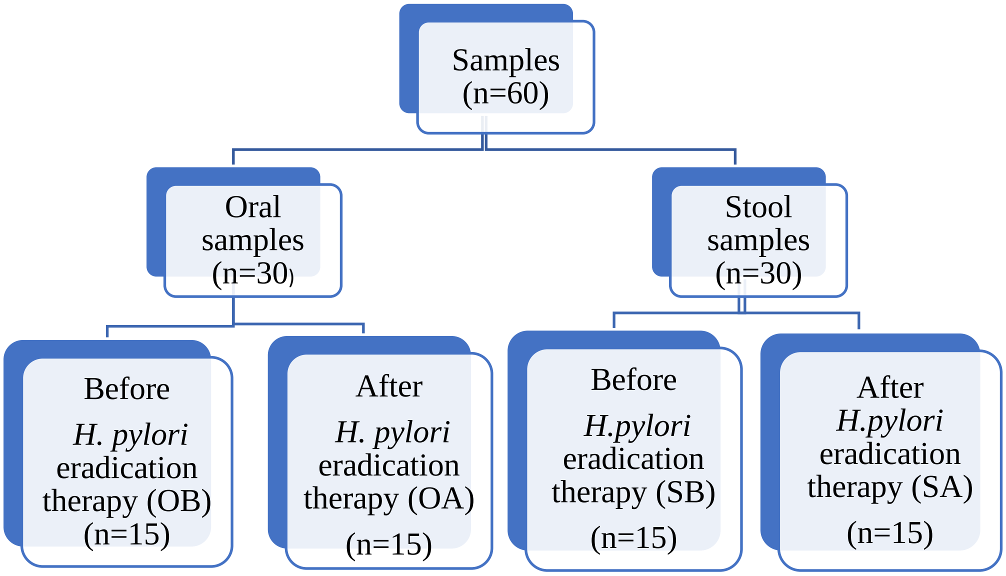

Supplementary Figure S1. The study design. A total of 60 salivary and stool samples collected from 15 participants; 15 oral samples before eradication (OB); 15 oral samples after eradication (OA); 15 stool samples before eradication (SB); 15 stool samples after eradication (SA).
